# Generation of Time-Series Working Patterns for Manufacturing High-Quality Products through Auxiliary Classifier Generative Adversarial Network

**DOI:** 10.3390/s22010029

**Published:** 2021-12-22

**Authors:** Manas Bazarbaev, Tserenpurev Chuluunsaikhan, Hyoseok Oh, Ga-Ae Ryu, Aziz Nasridinov, Kwan-Hee Yoo

**Affiliations:** 1 Elektrolitnyy Proyezd, 115230 Moscow, Russia; manasbazarbayev@gmail.com; 2 Department of Computer Science, Chungbuk National University, Cheongju 28644, Korea; teo@chungbuk.ac.kr (T.C.); garyu@chungbuk.ac.kr (G.-A.R.); 3 Department of Big Data, Chungbuk National University, Cheongju 28644, Korea; gyzmdh@chungbuk.ac.kr

**Keywords:** continuous casting machine, deep learning, induction furnace, time-series working patterns, auxiliary classifier generative adversarial network

## Abstract

Product quality is a major concern in manufacturing. In the metal processing industry, low-quality products must be remanufactured, which requires additional labor, money, and time. Therefore, user-controllable variables for machines and raw material compositions are key factors for ensuring product quality. In this study, we propose a method for generating the time-series working patterns of the control variables for metal-melting induction furnaces and continuous casting machines, thus improving product quality by aiding machine operators. We used an auxiliary classifier generative adversarial network (AC-GAN) model to generate time-series working patterns of two processes depending on product type and additional material data. To check accuracy, the difference between the generated time-series data of the model and the ground truth data was calculated. Specifically, the proposed model results were compared with those of other deep learning models: multilayer perceptron (MLP), convolutional neural network (CNN), long short-term memory (LSTM), and gated recurrent unit (GRU). It was demonstrated that the proposed model outperformed the other deep learning models. Moreover, the proposed method generated different time-series data for different inputs, whereas the other deep learning models generated the same time-series data.

## 1. Introduction

The origins of metal processing, which has become an important industry, can be traced back to the Bronze Age. The main piece of equipment in metal processing is a furnace—an enclosed structure intended for intense heating [1]. Numerous metal-processing methods have been developed. However, they were costly because charcoal was used as a fuel. Currently, two main furnace types are available: chemical and electric. In the former, melting is induced by combustion, whereas in the latter, it is induced by electricity. Electric furnaces were developed in the 19th century and are divided into electric-arc furnaces, in which an electrical current runs through electrodes inside the furnace, and induction furnaces, which use induction heating [2]. An electric-arc furnace uses the energy from the arc between the electrodes, whereas in an induction furnace, melting is caused by the heat produced by inducing a high-frequency current. In an induction furnace, the heat is generated by the excited molecules in the metal, and thus, whatever goes into the furnace is exactly what comes out. No oxygen or additional gases to the system are required, resulting in fewer variables to be controlled during the melting process [3]. Therefore, in this study, we focused on induction furnaces.

**Figure 1:**
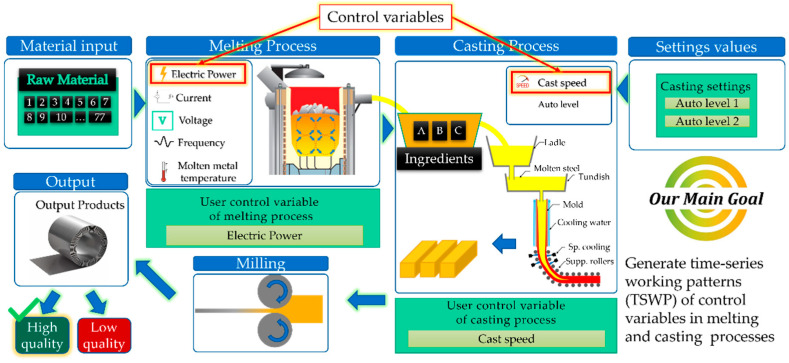
Overall process of metal production.

Figure 1 shows a flow chart of the metal production process. Here, the input is information about the material used to manufacture the final product, which is usually tabular data related to the amount of the raw material used. The selected amount of raw material is placed in the induction furnace, where it begins to melt. Then, the molten metal is poured into the casting, which produces slabs. The slabs are sent to the milling machine, which rolls them onto metal sheets or coils. In the end, the final products are checked by factory workers, and if their quality is poor, the previous steps are repeatedly applied until the poor-quality parts are removed. From the figure, we can also see that there are several control variables that are regulated by the operator. For example, the amount of electric power in the melting process and the casting speed in the casting process are control variables. We can collect control variables over time by monitoring the quality of the final product and use these variables to determine the working patterns of the manufacturing processes. In other words, these working patterns represent a set of optimal control variables that are needed to produce a good product. Considering that data related to the working patterns are collected at different points in time, we can call them time-series working patterns (TSWP).

Product quality is a major concern of manufacturers, as low-quality products cost labor, money, and time because they must be remanufactured. Therefore, determining the optimal working patterns is a key factor in producing high-quality products. Working patterns are not always constant but change based on product type and raw materials. Consider a typical metal manufacturing process, where an enormous amount of electric energy is consumed to melt the metal and make products. In this case, a great deal of time, money, and labor may be required to find the optimal user-controllable variables for machines and raw material compositions to produce a good product. On the other hand, we can produce pre-defined working patterns based on historical data from machines to help factories to avoid low-quality products and save labor, money, and time. Therefore, the main objective of this paper is to generate TSWP through AC-GAN for metal-melting induction furnaces and continuous casting machines, thus improving product quality by aiding machine operators.

There are many approaches to generate sequential pattern data, for example, Lee et al. [4] proposed a sequence pattern generation method using a sliding window technique. Moreover, the generation of time-series data using GAN-based models has been used in cyber-security [5], finance [6], and many others [7,8]. However, generating TSWP using GAN models in the manufacturing field has not been studied so far. Many studies have used GAN-based models in the manufacturing field; however, most cases have focused on detecting defect products based on image datasets [9,10] and non-time-series datasets [11,12,13]. This study uses historical data from melting and casting processes to generate TSWP using a generative deep learning approach. More specifically, the main contributions of this study are as follows:

- We first defined a TSWP dataset. The TSWP dataset was generated by integrating various information related to the melting and casting processes, such as material weights, ingredient percentages, controllers (i.e., electric power, current, voltage, etc.), and time measures because each process consists of different steps.
- We then applied several data preprocessing techniques to prepare the dataset for the training and testing of the proposed model. The proposed TSWP dataset has different characteristics. First, the number of features in the TSWP dataset is different in each row because the duration of the process varies. To solve this problem, we expanded the data using the maximum number of features. Second, we applied the data normalization technique to solve the large variations in the TSWP dataset. Third, we transformed some categorical features into numerical features. Lastly, we filtered the data for high-quality products to generate TSWP only for such products.
- We used the AC-GAN method to generate the TSWP based on the historical data from the melting and casting processes. This method consists of two models: a generator and a discriminator. First, the discriminator is trained by a batch of actual data from the training set and an equal number of synthetic data points from the generator. Then, the generator produces another batch of data, and the discriminator determines whether the data are actual or synthetic.
- Lastly, we trained and evaluated the AC-GAN method and other deep learning methods: MLP, CNN, LSTM, and GRU. The experiments demonstrated that the proposed method has two advantages over the other deep learning methods: (1) it dramatically reduces the error rate, and (2) it generates different outputs for different inputs.

The rest of the paper is organized as follows. In Section 2, related work is reviewed. Section 3 describes the overall procedure in detail, explains the collection of data, their preparation and preprocessing steps, and elaborates on the AC-GAN method, whereby TSWP are generated. In Section 4, the experiments are presented. Finally, the paper is concluded in Section 5.

## 2. Related Work

Studies on metal processing have focused on, for example, defect prevention, production forecasting, and maintenance prediction. We can classify these studies into the following three categories: (1) studies that use statistical methods, (2) studies that use machine learning methods, and (3) studies that use deep learning methods. The subsequent sections discuss each category in detail.

### 2.1. Statistical Methods

Several studies have used statistical methods to analyze metal processing [14,15,16]. For example, Adetunji et al. [14] investigated the relationship between the energy consumption of a molten bath and its chemical composition in steelmaking. The authors used numerical modeling to predict the melting time in induction furnace operation. They also provided insights into the reduction of energy consumption and estimated production time using material charge balancing. The results from their model prediction and real-time melting exhibited highly satisfactory performance with an overall accuracy between 81% and 95%. Ean et al. [15] proposed a method for analyzing and determining the working patterns in nonferrous electric arc-furnace plants by adopting dynamic programming. To determine the best objective value candidates, statistical methods were used to obtain the optimal total elemental power and total product quantity. The authors searched for efficient working patterns for a logical concept called the tap position. The tap position is the control variable of the electric arc furnace, which heats the raw materials by using the electrodes. They combined the consumed electric power and produced product quantity to select the best candidate between the previous, current, and next steps using a dynamic programming approach. Kovačič et al. [16] analyzed the electrical energy consumption during the melting, refining, and tapping process in arc furnace operation at the Štore Steel company. The authors employed two kinds of statistical methods: linear regression and genetic programming modeling. There were 3248 consecutively produced batches with 25 features (i.e., coke, dolomite, and quantity in melting, injected oxygen, and carbon in refining and tapping) in 2018 for creating models. The authors validated the models using 278 batches produced in 2019. The experimental results showed that it was possible to reduce the average electric energy by 1.04% and 1.16% in linear regression and the genetically developed model, respectively.

### 2.2. Machine Learning Methods

Many studies have used machine learning methods such as decision trees (DT), random forest (RF), and support vector machines (SVM) [17,18,19,20,21]. Karunakar and Datta [17] predicted major casting defects using a backpropagation neural network collected from a steel producer. The four mold properties and the seven melting parameters were used as input and the nature of the castings (valid or defective) as output for the neural network, which used one hidden layer with 23 neurons. They reported the effectiveness of using an artificial neural network (ANN) for defect prediction because factory workers are warned when a defective casting is about to be manufactured. Hore et al. [18] outlined the application of a data-driven MLP-based ANN model to characterize the effects of melt composition, tundish temperature, tundish superheat, casting speed, and mold oscillation frequency on important processing parameters and to predict the occurrence of defects in the cast product. The chemical composition of the melt was used as the input of the model, and the average values of tundish temperature, tundish superheat, cast speed, and mold oscillation frequency were recorded at four time intervals. The oscillation depth, mold powder consumption rate, metallurgical length, and probability of crack occurrence were the output of the model. It was demonstrated that the prediction results of the proposed method were quite satisfactory.

Ye et al. [19] proposed a weighted RF method to predict the quality of the casting process based on multi-process parameters in real-time. This method solves an imbalanced classification problem by using a weighted DT. The authors mentioned that the proposed method could correctly identify negative samples. The experimental results demonstrated the effectiveness of the proposed method in real-time production. Lee et al. [20] predicted the quality of the metal casting process and operation control using several machine learning algorithms, such as DT, RF, ANN, and SVM. The authors trained machine learning models by developing a cyber-physical production system dashboard—a virtual representation of a real-world manufacturing process. Dučić et al. [21] presented an application that controlled the alloying process of white cast iron production. They used neural network and Support Vector Regression (SVR) models to predict the amount of alloying additives using the desired chemical composition of white cast iron. Three hundred melting batches were used to train the models. The data were organized into four groups, each of which had four chemical compositions. The first group consisted of 2.5 tons of molten metal chemical composition that remained in the furnace after the melting process. The second group consisted of the chemical composition of 5 tons of steel waste added into the furnace. The third group represented the chemical composition of the melted iron after the alloying process. Finally, the fourth group of data represented the amount of alloying additives added during the alloying process. The testing results demonstrated that both the neural network and the SVR model are suitable and reliable for the control of the alloying process during white cast iron production. Moreover, the paper revealed that the neural network model could be a powerful tool for controlling activities related to the metal melting process.

### 2.3. Deep Learning Methods

Deep learning approaches have been applied to the metal casting process, as with other manufacturing applications [22,23,24,25]. For example, Lee et al. [22] proposed a spatial and sequential deep learning approach to predict temperature distribution during the casting process. The authors claimed that casting quality is highly dependent on temperature distribution, and they proposed a model for predicting this distribution by integrating a CNN and a recurrent neural network (RNN). The experimental results indicated that the proposed model outperformed the baseline models and were applied to the real-world industry by providing real-time temperature prediction. Lee et al. [23] proposed a fault-detection method based on one-class deep learning for imbalanced data. The data were based on the temperature for an actual metal molding process at seven locations, consisting of 1430 success and 70 failure cases. The authors used four different prediction models: MLP, residual network (ResNet), LSTM, and ResNet–LSTM. The experimental results demonstrated that the one-class classification model improved the accuracy of the baseline models from 90% to 96%. This study suggests that the one-class deep learning approach is suitable for imbalanced manufacturing fault detection. Song et al. [24] proposed a hybrid deep learning model consisting of a CNN and a deep neural network (DNN) to avoid faults in the continuous casting process. The dataset consisted of input features (i.e., cooling water temperature values) and output features (i.e., surface temperature, ambient temperature, initial temperature, casting speed, thickness, and width). The authors collected data from 32,678 samples with 15 variables. The experimental results indicated that the proposed method overcame the limitations of state-of-the-art models. The authors also mentioned that the proposed model could reduce computational time and prediction error. Habibpour et al. [25] developed a deep learning framework to detect defects in casting products. The framework consists of feature extraction and product classification. First, the authors employed four types of CNN-based models (i.e., VGG16, ResNet50, DenseNet121, and InceptionResNetV2) to extract meaningful features from the casting image dataset. The dataset contains 1300 samples of 512 × 512 grey-scaled image data: 781 images with defects and 519 non-defect images. Second, the authors performed the classification task using SVM and MLP based on the extracted features. The experimental results showed that the VGG16 algorithm outperformed the other three algorithms. The authors declared that their solution on defect detection would reinforce the quality assurance of casting productions.

### 2.4. Discussions

Product quality is a major concern in many manufacturing industries. For example, in the metal processing industry, low-quality products must be recycled, which requires additional labor, money, and time. This section has discussed several studies that focused on defect prevention, production forecasting, and maintenance prediction in the metal processing industry using statistical, machine learning, and deep learning techniques. Unlike the existing studies in this paper, we propose the AC-GAN model to generate the optimal working patterns based on the historical data from melting and casting processes. Although some studies used GAN-based models in the manufacturing field, most have focused on detecting defect products based on image datasets [9,10] and non-time-series datasets [11,12,13]. In other words, generating working patterns using GAN models in the manufacturing field has not been studied so far. Recall from Section 1 that TSWP represents a set of optimal control variables and raw material compositions needed to produce a good product. However, the main challenge is that a great deal of time, money, and labor may be required to find the optimal user-controllable variables for machines and raw material compositions to produce a good product. Therefore, this paper proposes a method that generates pre-defined TSWP based on historical data from machines to help factories avoid low-quality products and save labor, money, and time.

## 3. Materials and Methods

### 3.1. Overview

**Figure 2:**
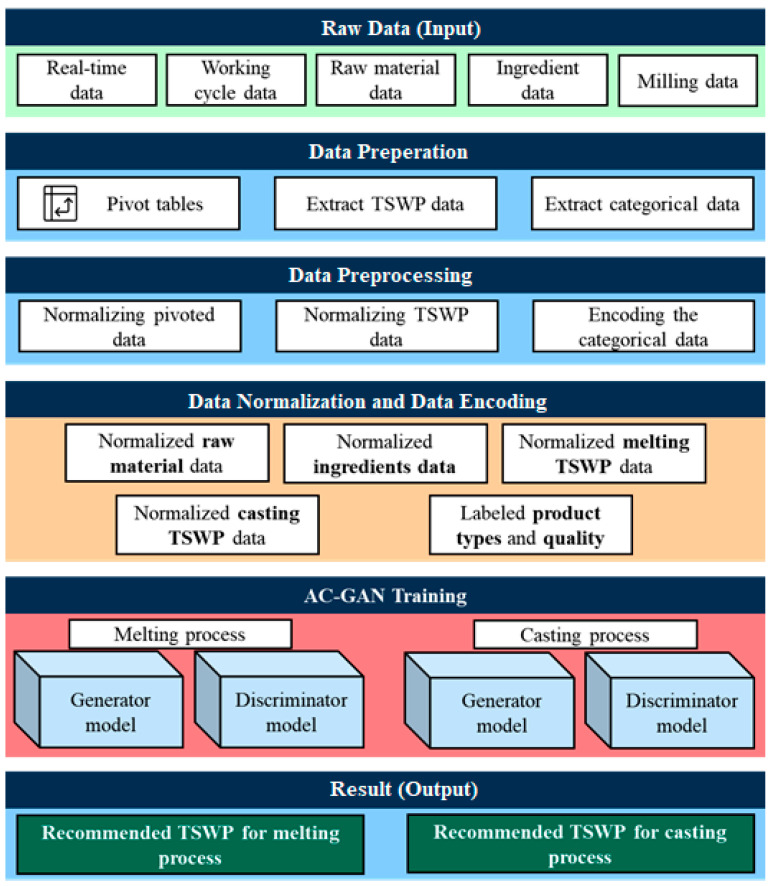
Overall process of the proposed method.

The overall process flow of the proposed method is shown in Figure 2. It consists of five steps. First, raw data are obtained from the server. Second, the raw data are transferred to the preprocessing step, where raw material and ingredient data are pivoted, and categorical data and TSWP are extracted using working cycle data. Third, pivoted and TSWP data are normalized, and categorical variables are encoded. Fourth, the prepared data are passed to the corresponding models for training. The last step involves the concept of the AC-GAN model, where the generator receives the latent space, product type, and raw-material data or ingredient data, depending on the process, and then outputs the respective TSWP pattern. The discriminator receives real data or generated data as input and outputs the probability that the data are real or generated and the product type. We describe each step in detail in the following subsections.

### 3.2. Production Process

**Figure 3:**
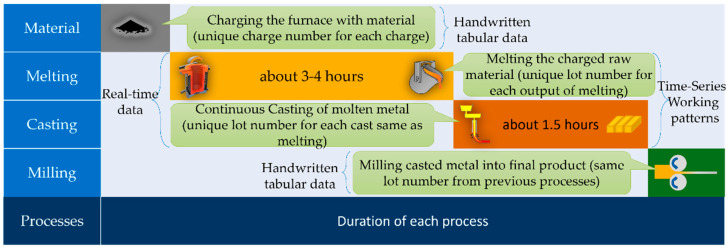
Overall process of metal production.

Figure 3 demonstrates the overview of the manufacture of metal products, which consists of several steps. First, a raw material mixture is required, which is melted in the induction furnace, and a report about used raw materials is written by workers and stored in the database as tabular data. Then, the induction furnace, controlled by an operator, begins to melt the raw materials. During the melting process, the electric-energy-consumption data are read from the programmable logic controller (PLC) in the factory. The melting process data are stored as time-series data because the PLC sends them to the server in real-time every second. When the metal becomes liquid, the workers inspect the percentage of the ingredients of the molten metal. If the requirements are not met, melting should be continued. The ingredient data are also recorded by workers and stored in the database as tabular data. If the ingredients satisfy the requirements, the molten metal is poured into a continuous casting machine, solidifying it into a semi-finished slab. An operator also monitors the continuous casting machine during the casting process, which sends time-series data to the server. After the casting process, the produced slabs are passed to a milling machine that converts them into coils. If the milling process has been performed only once, the product is of high quality; otherwise, the final product is of low quality and has been processed several times. The milling process report is also recorded by workers and stored in the database as tabular data. The TSWP can be extracted from the working cycle data, which contain information about the beginning and end of production processes. TSWP may differ depending on the type of production. Hence, to generate TSWP for manufacturing high-quality products, the data should be prepared to fit the models. As there are two types of processes, two different models are required for each process due to the different durations.

### 3.3. Problem Statement

**Figure 4:**
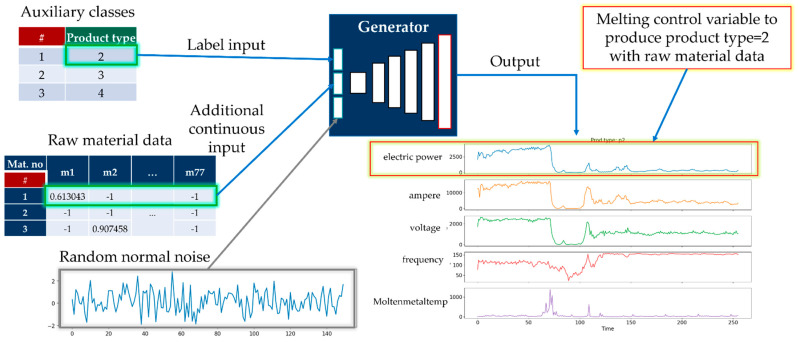
The generator model of AC-GAN for the melting process.

In this paper, we generate TSWP through AC-GAN historical data from melting and casting processes. The AC-GAN [26] is the extension of GAN [27], consists of a generator model and discriminator model. The generator model takes class labels, random points from the latent space, and existing real data to generate new synthetic data. On the other hand, the discriminator model receives generated or existing data as input to distinguish it from real or synthetic data. We generate TSWP data based on production process types (i.e., melting and casting) using AC-GAN. Considering that we have two different processes that depend on TSWP, we construct two AC-GAN models for each process. The implementation details of the generator and discriminator models for both melting and casting processes are given in Appendix A. Figure 4 shows the generator model with a sample input and generated output. The generator model of the AC-GAN of the melting process has the following inputs:

- Auxiliary class input, product type of working cycle data;
- Auxiliary continuous input from raw material data;
- Latent space data (random normal) of length 150.

The output of the generator model for the melting process is a generated TSWP. The most important variable in the output TSWP is the electric power control variable because, by following this pattern, high-quality products can be manufactured. The generator model consists of three input layers. The first is the product-type label layer, with 11 units because there are 11 distinct product types. The second is the material information layer, with 77 units because there are 77 variables in the material input tables. The third is a latent space that contains random noise with selected shapes; in our case, the shape is 150. As the input layers have different shapes, we should first pass the data to the dense layers with the same units and reshape them into the same shapes.

**Figure 5:**
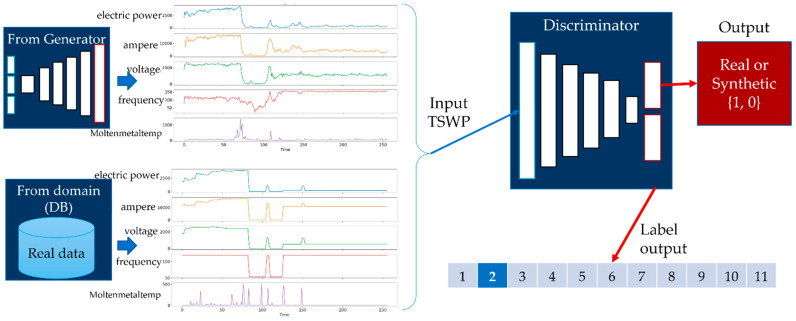
The discriminator model of AC-GAN for the melting process.

For the discriminator model, the input is the generated TSWP of the melting process. The input shape for the discriminator model is 256 × 5, where 256 indicates the duration of the process and 5 is the number of features of the process. The discriminator model has two outputs: the first is the probability that the input is real, which implies that the discriminator attempts to distinguish real data from generated data, and the second is the auxiliary class of the input data, which, in our case, is the product type. The discriminator receives both real and generated data during training, with a label of 1 for real data and 0 for generated data, as shown in Figure 5. The goal of the generator model is to generate TSWP data that cannot be distinguished from real data by the discriminator. If the discriminator cannot distinguish the generated data from the real data, it can be concluded that the generator generates similar data to real data.

**Figure 6:**
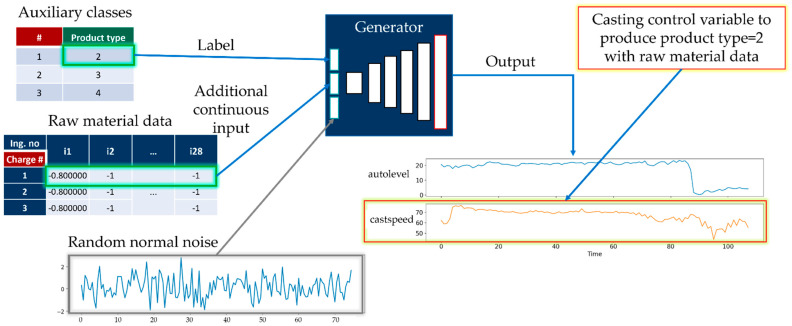
Generator model of AC-GAN for the casting process.

Figure 6 shows the generator model with sample input and generated output. In the case of the casting process, the generator model consists of the following inputs:

- Auxiliary class input, the product type of working cycle data;
- Auxiliary continuous input from ingredient data;
- Latent space data (random normal) of length 80.

In this case, the output of the generator model for the casting process is a generated TSWP of the casting process. The difference between the melting and casting processes is the shape of the TSWP for each process. Thus, we constructed different models for each process. The generator of the casting process also consists of three input layers: the first is a product type layer with 11 units, as in the melting process; the second is ingredient information, which has 28 units because the ingredient table has 28 variables, and the third is a latent space with random noise and a shape of 80. In this case, the input layers are passed to the dense layers with the same units and then reshaped to the same shape.

**Figure 7:**
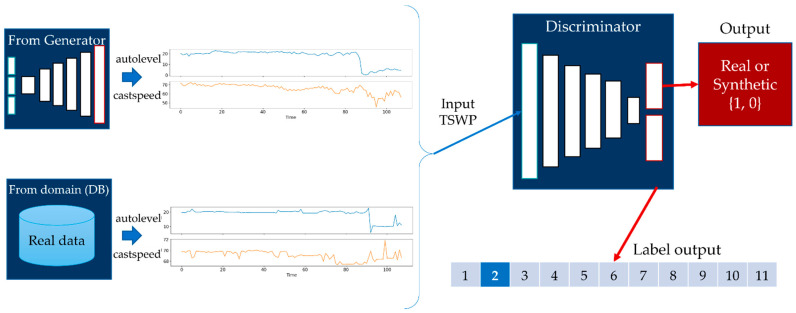
Discriminator model of AC-GAN for the casting process.

For the discriminator model, the input is the generated TSWP of the casting process. The input shape for the discriminator of the casting process is 108 × 2, where 108 indicates the duration of the casting process, and 2 represents the number of features of the casting process. In this case, the discriminator also has two outputs: the probability that the input data are real, and the auxiliary class of the input data, as shown in Figure 7. During the training step, the discriminator receives both real data, which are labeled with 1, and generated data, which are labeled with 0. The goal of the generator model is to generate a TSWP of the casting process that cannot be distinguished from real data by the discriminator.

### 3.4. Dataset

#### 3.4.1. Data Preparation

As we mentioned in Section 1, we use several raw data types as input to our model, including real-time data, working cycle data, raw material data, ingredient data, and milling data. First, the PLC transmits the data from the sensors in real-time every second. These real-time data are collected from the database and aggregated per minute. Sample rows from the real-time data are given in Table 1. The time variable is used as the index variable. The electric power variable corresponds to the primary user control variable of the melting process, and the current, voltage, and frequency variables correspond to machine variables related to electric induction in the induction furnace at the indicated time. Molten metal temperature is the temperature of the melted metal inside the furnace. Auto level 1 and auto level 2 are machine variables, and the cast speed variable is the control variable of the casting process. In addition, in the table, the units of electric power, current, voltage, frequency, molten metal temp. and cast speed are kilowatts (kW), ampere (A), volt (V), hertz (Hz), the temperature in Celsius (°C) and meters per minute, respectively. The real-time data comprise 802,569 records from 28 April 2019 to 1 January 2021.

Second, the working cycle data are manually recorded by factory workers and contain information about the production process. Specifically, working cycle data consist of variables such as Lot#, Charge#, product type, Mstime, Metime, Cstime, and Cetime. Here, Lot# is a unique value for each product that is used for linking with other tables. Charge# is a value that defines the charge of materials in the induction furnace and is also used for linking. Product type indicates encoded information about a product; there are 11 products in total, encoded as {p1,…,p11}. Mstime is the melting process start time, and Metime is the end time of the melting process. Similarly, the Cstime and Cetime variables store information about the start and end times of the casting process. Using this information, it is possible to extract and filter other data for our problem.

Third, information about raw materials, which are melted in the induction furnace, are manually recorded. The raw materials data consist of variables, such as Charge#, material number, and weight. Here, the Charge# variable is used for linking with other data. The material number variable has categorical values encoded for materials used in the melting process. Specifically, there are 77 materials in total, encoded as {m1,…,m77}. For each record with a unique Charge#, there are 77 variables of materials, where the unused materials have zero values. All records correspond to the same Charge#, implying that different materials are put into the furnace for the same Charge#. The weight variable is the weight in kg of each material.

Fourth, ingredient data are also manually recorded by workers when they check the molten metal. The ingredient data have a structure similar to the raw material data. Specifically, ingredient data consist of variables, such as Charge#, ingredient number, and weight. The Charge# variable is used for linking with other data. The ingredient number variable has categorical values that represent explored ingredients after melting; there are 28 ingredients in total, encoded as {i1,…,i28}. The percentage attribute is the percentage of each explored ingredient. For each row (i.e., for each unique Charge#), there is a percentage (%) value corresponding to each variable, which denotes a unique ingredient type.

Fifth, milling data contain information about the number of millings. Milling data consist of variables such as Lot#, Charge# and Milling#. As before, the Lot# and Charge# variables are used for linking with other data. Milling# is the number of millings of the final product. It is the key variable for defining the quality of the final product, which is calculated according to Equation (1):

(1) $${\mathrm{Quality}}_{i}=\{\begin{array}{c} \mathrm{High},\;Milling_{i}=0\;\\ \mathrm{Low},\;\mathrm{otherwise}\; \end{array}$$

Based on data from the production process described above, it is possible to extract TSWP. As shown in Table 1, the real-time data are indexed by the time variable, and we also have working cycle data, which contain information about the start and end times of the melting and casting processes. Therefore, we can use the Mstime and Metime variables to extract the TSWP of the melting process and the Cstime and Cetime variables to extract the TSWP of the casting process. For the melting process, we should extract five variables from the real-time data: electric power, current, voltage, frequency, and molten metal temperature. Table 2 shows a sample of the extracted TSWP data for Charge# 1 and Lot# 1 for the melting process. The first six variables in this table can be explained as follows. For each record with time t, the induction furnace used electricity with electric power, current, voltage, frequency values shown in each cell, and molten metal temperature was recorded for this t. Additional information, such as Charge# and Lot#, is used for filtering operations.

Table 3 shows a sample of the extracted TSWP data for Charge# 1 and Lot# 1 for the casting process. For the casting process, two variables should be used from real-time data: auto level and cast speed. Auto level will be combined depending on the Lot# value. If it is an odd value, it should be auto level 1; otherwise, it should be auto level 2. Furthermore, additional information such as Lot# and Charge# will be added to the extracted TSWP of each process.

**Figure 8:**
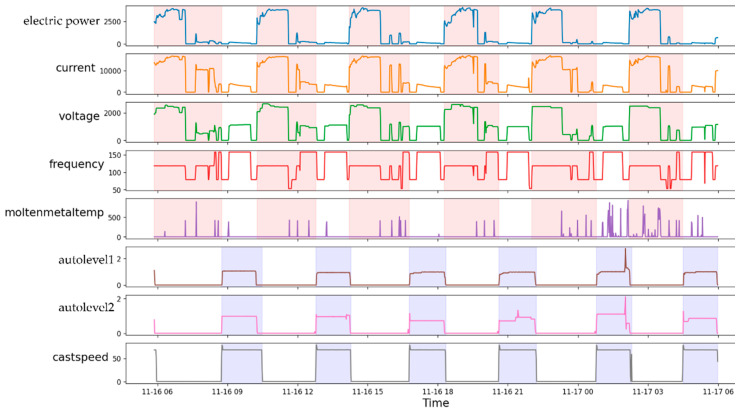
Extracting melting and casting TSWP from real-time data (pink areas indicate melting process, purple areas indicate casting process).

Figure 8 shows the real-time data with the extracted TSWP, where the pink areas indicate the TSWP of the melting process, and the purple areas indicate the TSWP of the casting process. The first three variables indicate that for each value of time, the casting machine used the auto level and cast speed values shown in the cells. As can be seen in the plot, cast speed, which is the control variable of the casting process, has only zero values when the melting process is in progress. In contrast, when the casting process is in progress, electric power, which is the control variable of the melting process, has almost zero values. From Figure 8, it can also be seen that the duration of each TSWP may be different, particularly in the areas with pink color, and it can also be challenging to find a constant value for the duration of each process.

**Figure 9:**
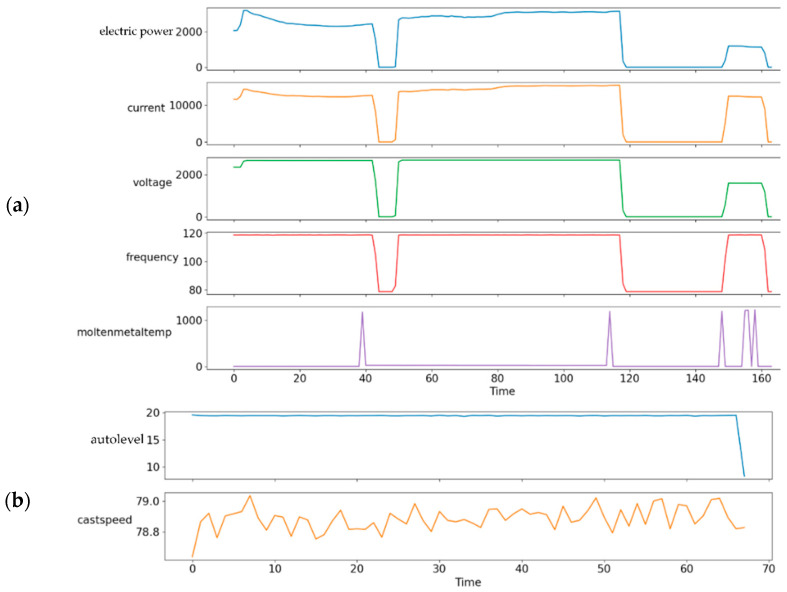
Detailed visualization of extracted TSWP: (**a**) melting process and (**b**) casting process.

The detailed visualizations of the TSWP of the melting and casting processes are shown in Figure 9, where it can be seen that the melting and casting processes are different. Specifically, they have different features and durations. In the case of the melting process, there are five features with a duration of 163, and for the casting process, there are two features with a duration of 69. Considering that there are two types of processes, two different models are explained in Section 3.3 for each process due to the different durations.

#### 3.4.2. Data Preprocessing

Data expansion. As noted previously, the duration of the processes varies. Specifically, we analyzed the duration of each TSWP of the melting and casting process and found that the maximum duration for the melting process is 256 min, and that of the casting process is 108 min. However, the models require the same shapes for each input and output, and thus they should be adapted to the same durations. To prepare equally shaped inputs, we expanded each process to the maximum minutes used in processes using Algorithm 1. Here, *N* is the maximum minutes in processes (i.e., 256 min in melting and 108 min in casting processes) and *D* is the list of processes. Algorithm 1 expands a process by copying the last row until the process is reaches its maximum number of minutes.
**Algorithm 1.** **Expanding data into length *n*.**1 2 3 4 5 6 7 8 9**INPUT:** *N* ← desired length *list D* ← list to expand. *last_value* ← Last value of list D. **OUTPUT:** *expanded_list_D* **for**
*n-len(D)*
**do** Append *last_value* to *D.* **end for**

**Figure 10:**
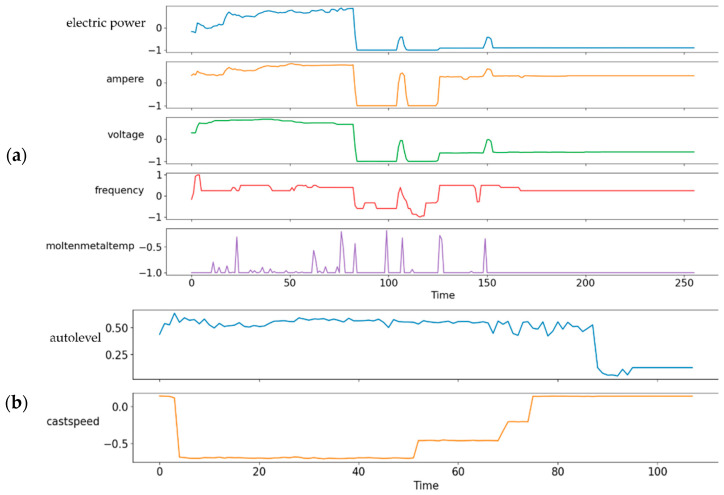
Result of application of min–max normalization to TSWP data: (**a**) melting process and (**b**) casting process.

Data normalization. Data normalization is one of the essential preprocessing methods for machine learning and deep learning techniques. An appropriate normalization of the input data before model training reduces estimation error and training time. Our data analysis in Table 4 reveals that the statistical measures (i.e., maximum, mean, and standard deviation) of the data vary, meaning that the data must be normalized. The min–max normalization method is used to rescale the features between −1 and 1. The result of the application of min–max normalization to TSWP data for the melting and casting process can be seen in Figure 10.

Data encoding. Machine learning and deep learning models often require the conversion of categorical data into numeric representations. In most cases, data encoding is required because the models cannot handle categorical or string data. Thus, data encoding is one of the most widely used methods to accomplish this. A data encoder simply assigns an integer to every possible value of a categorical variable. The Python Scikit-learn library provides a Category Encoder module, in which LabelEncoder encodes labels with a value between 0 and the number_of_classes–1, where number_of_classes is the number of distinct labels. If a label is repeated, it is assigned the same value. The dataset that we study in this paper also requires data encoding. For example, the working cycle data have a categorical variable in the Product type column, the classes of which are text values: {p1,…,p11}. These values are encoded using LabelEncoder as {p1,…,p11} 0,…,10. As a result, in the working cycle data, there are now encoded product types with values in the range 0 to 10.

Data filtering. The main goal of the paper is to generate TSWP only for high-quality products. Considering that the proposed model should be trained with historical data related to high-quality products, the input data should be filtered depending on product quality, determined by the milling data. Thus, we used data filtering procedure to prepare high-quality products for training data. The input to the data filtering procedure is a list of raw data (i.e., real-time data, working cycle data, raw material data, ingredient data, and milling data) that includes both high-quality and low-quality products. The data filtering procedure first filters out milling data with Milling# equal to the 0 condition. Then, the other table indexes are filtered using the Lot# and Charge# values of the filtered milling data depending on their index column. After all the preparation and preprocessing steps, the data can be used for training.

### 3.5. Generation of Time-Series Working Patterns Using AC-GAN

Algorithm 2 explains the AC-GAN model in more detail based on its cost functions. As we mentioned in Section 3.3, the AC-GAN model requires two models: a generator and a discriminator. In addition, there are two types of processes, namely the melting and casting processes, and for each process, we created AC-GAN models. The input of the generator model for the melting process consists of a latent space (random normal noise), product type, and one row from the raw material data. The output of the generator is a synthetic TSWP of the melting process with the same shape as shown in Figure 10a. The discriminator model of the melting process uses real data from the domain or synthetic data from the generator as input, and it outputs the probability that the input data are real and the categorical data that describe the product type of the input data. For the casting process, the input of the generator model also consists of a latent space and product type. However, instead of the raw material data, the ingredient data are used as additional input, and the output is a synthetic TSWP of the casting process, with shapes as shown in Figure 10b. The discriminator of the casting process receives real input data from the domain or synthetic data from the generator model, and the output for these data is the same as the previously mentioned probability and product type. In Algorithm 2, the two log-likelihood functions, $L_{s}$ and $L_{c}$, for correct source and correct class in AC-GAN are given as Equations (2) and (3) [26,28]:

(2) $$L_{s}=\frac{1}{m}\overset{m}{\underset{i=1}{\sum }}\left[\log\;D\left(x^{\left(i\right)}\right)+\log\left(1-D\left(G\left(z^{\left(i\right)}\right)\right)\right)\right].$$

(3) $$L_{c}=\frac{1}{m}\overset{m}{\underset{i=1}{\sum }}\left[P\left(class=c^{\left(i\right)}|x^{\left(i\right)}\right)+P(class=c^{\left(i\right)}|G\left(z^{\left(i\right)}\right))\right].$$

where *x*, *z*, and *c* are real, noise and class data, respectively and *m* is minibatch size. In AC-GAN, discriminator *D* (generator *G*) is trained to maximize $L_{s}$ + $L_{c}$ (resp. $L_{c}$ − $L_{s}$).
**Algorithm 2. AC-GAN**
**INPUT:**1*n* ← number of training iterations2*k* ← number of steps3*m* ← minibatch size4*z*^(i)^← noise data5*x*^(i)^← real data6*c*^(i)^← class data7η← learning rate8**OUTPUT:**9*generated_TWSP*
**Initialize:** discriminator *D* with parameter $\theta_{d}$ and generator *G* with parameter $\theta_{g}$
10**for** *N* **do**11**for** *k* steps **do**12Sample minibatch of *m* noise samples {*z*^(1)^, …, *z*^(*m*)^} from noise prior *p_g_*(*z)* with class labels {*c*^(1)^, …, *c*^(*m*)^}13Sample minibatch of *m* examples {*x*^(1)^, …, *x*^(*m*)^} from data generating distribution *p*_data_(*x*).14Update $\theta_{d}$ by maximizing $L_{c}+L_{s}$:15$\theta_{d}=\theta_{d}+\eta \nabla \theta_{d}\left(L_{c}+L_{s}\right)$(4)16**end for**17Sample minibatch of *m* noise samples {*z*^(1)^, …, *z*^(*m*)^} from noise prior *p_g_*(*z)*.18Update $\theta_{g}$ by maximizing $L_{c}-L_{s}$:$\theta_{g}=\theta_{g}+\eta \nabla \theta_{g}\left(L_{c}-L_{s}\right)$(5)19**end for**

### 3.6. Methods under Comparison

In this paper, we applied five deep learning methods to generate TSWP for a selected product, thus aiding factory workers to manufacture high-quality products. These methods are AC-GAN, MLP, CNN, LSTM, and GRU. Table 5 lists the parameter settings for these methods. We implemented the methods using several combinations of parameters and selected the best options.

The first model is ANN. An ANN is a concept inspired by networks of biological neurons in the brain [29]. MLP is a basic type of feed-forward ANN. It comprises at least three layers: an input layer, hidden layers, and an output layer. DNN has more hidden layers. Each layer consists of a defined set of neurons that are fully connected to the next layer. The neural network updates the weights of these connections using a backpropagation algorithm [30]. Lara-Benítez et al. [31] mentioned a series of studies that used MLP for different time-series forecasting problems, which yielded better results than statistical methods. CNN is a type of DNN that was inspired by the visual cortex of the brain. The first CNN, a special ANN architecture, was proposed by LeCun et al. in 1988 [32]. Originally, CNN was designed for computer-vision tasks. It involves convolution operations, the essence of which is that each fragment of an image is element-wise multiplied by the convolution matrix (kernel), and the result is summed up and written to a similar position in the output image.

An RNN is a deep learning model that predicts the current value by looping past information. The GRU and LSTM models overcome the limitation of RNN by utilizing additional gates to pass information in long sequences. Specifically, a GRU autoencoder is another version of an autoencoder for sequential data using an encoder–decoder based on the GRU architecture [33]. The GRU cell uses two gates: an update gate and a reset gate. The update gate determines whether to update a cell. The reset gate determines whether the previous cell state is important. An LSTM autoencoder [34] is a version of an autoencoder model for sequential data using an encoder–decoder based on the LSTM architecture [35]. The LSTM cell uses three gates: an insert gate, a forget gate, and an output gate. The insert gate is the same as the update gate of the GRU model. The forget gate removes the information that is no longer required. The output gate returns the output to the next cell states.

**Figure 11:**
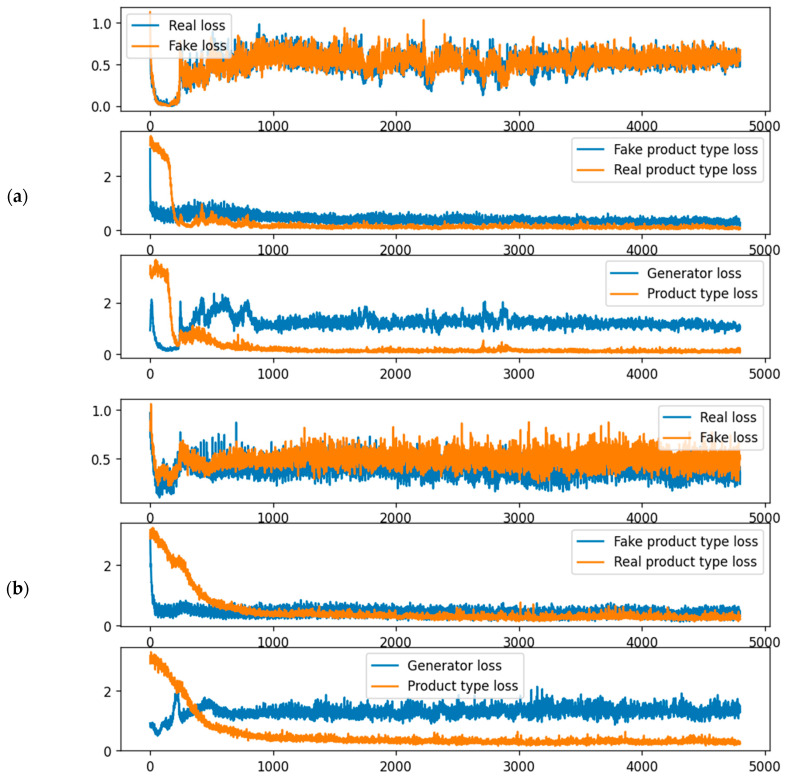
Training loss values of AC-GAN: (**a**) melting process and (**b**) casting process.

In this paper, we use the AC-GAN model to generate TSWP data. The AC-GAN models were implemented using the Keras module of the TensorFlow 2.0 library. The loss values during the training of the AC-GAN for the melting process are shown in Figure 11. The top of the figure shows the loss of the discriminators’ first output for real and synthetic data. It can be seen that the losses are close to 0.5, implying that a Nash equilibrium is almost reached. In addition, the discriminator cannot distinguish the synthetic data, implying that the generator is effective. The second plot is the loss of the second output of the discriminator, which is used to classify the product type. Its values are close to 0, implying that it can distinguish the product type of the input TSWP for the melting process. The last plot shows the generator loss and the product type loss for only the synthetic input.

Originally, the main goal of the GAN-based methods is to generate new believable synthetic data by learning a set of training data. The newly generated data are used for several purposes in the manufacturing process: (1) to create training data when data are insufficient and costly to collect, (2) to detect defective products, and (3) to set optimal control values of the manufacturing machines. In particular, the GAN-based methods have more advantages than other deep learning methods when it comes to the third purpose. Specifically, other deep learning models, such as CNN and LSTM, are not designed to generate new complex data, but are mostly suitable for classifying or predicting single or multiple values. On the other hand, according to the architecture of the AC-GAN, we can simply generate TSWP of the same characteristics as the currently limited TSWP data we have. For example, we can find the optimal control values of the melting machine using product type and raw materials. To obtain similar results, other deep learning models should involve creating a large dataset and performing complicated processing steps. In other words, the difference between GAN models and autoencoders is that a GAN has a generative architecture, whereas autoencoders have a predictive architecture. The training processes are different for these models because autoencoders do not have two models for training, as is the case with GAN. Therefore, the AC-GAN method fits our purpose, which is to generate TSWP based on product type and materials. More specifically, we assume that the AC-GAN method can simplify the complex process of setting optimal control values for manufacturing machines.

## 4. Results

In this section, we demonstrate the results of experiments. Specifically, we first present the efficiency of the proposed AC-GAN model compared with other deep learning models in terms of accuracy and processing time in Section 4.1, and then we present a visual comparison of the results in Section 4.2.

### 4.1. Evaluation

**Figure 12:**
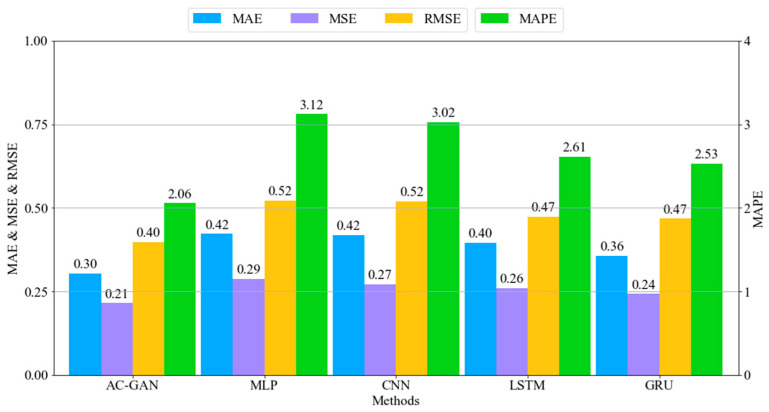
Error rates of melting process models.

**Figure 13:**
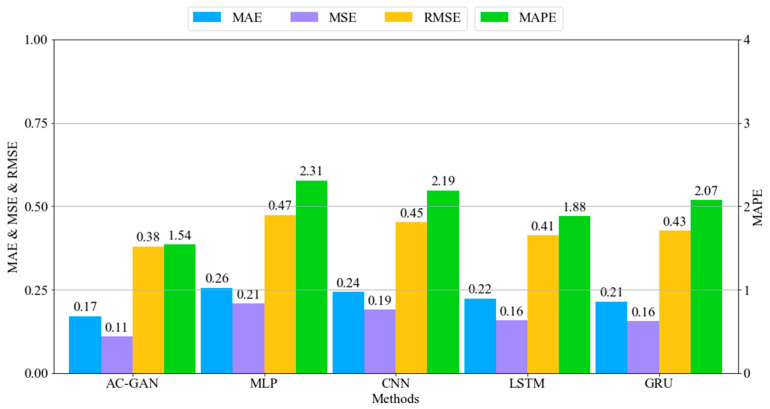
Error rates of casting process models.

After training all the models, we first compared the accuracy of the competing models. Specifically, the five neural network models—AC-GAN, MLP, CNN, LSTM, and GRU—were validated by using the mean absolute error (MAE), mean squared error (MSE), root mean squared error (RMSE), and mean absolute percentage error (MAPE). To evaluate the predicted or generated data from the networks, we compared the results with ground-truth data from the domain. We generated 4228 working cycle data points, among which 3376 high-quality working cycle data points were selected for our evaluation. The selected working cycle data were split into sets of 2282 and 1094 for train and test, respectively. Specifically, the splitting ratio of training and testing data was 67% for training and 33% for testing. Figure 12 and Figure 13 show the metrics for the melting and casting processes, respectively. It can be seen that the AC-GAN models perform better than the other deep learning models for both the melting and casting processes. More specifically, the AC-GAN model of the melting process outperformed the other deep learning models by around 17.5% to 30% in terms of RMSE. In the case of the casting process, the AC-GAN model outperformed the other deep learning models by around 7.9% to 23.7%. According to the demonstrated results, we can conclude that the proposed AC-GAN model is accurate in generating TSWP.

Next, we compared the training time for each model and each process. Table 6 shows a comparison of the execution time of all the models. The long training process of AC-GAN is because two models are trained, whereas the other models have only one model.

### 4.2. Visual Comparison of Results

**Figure 14:**
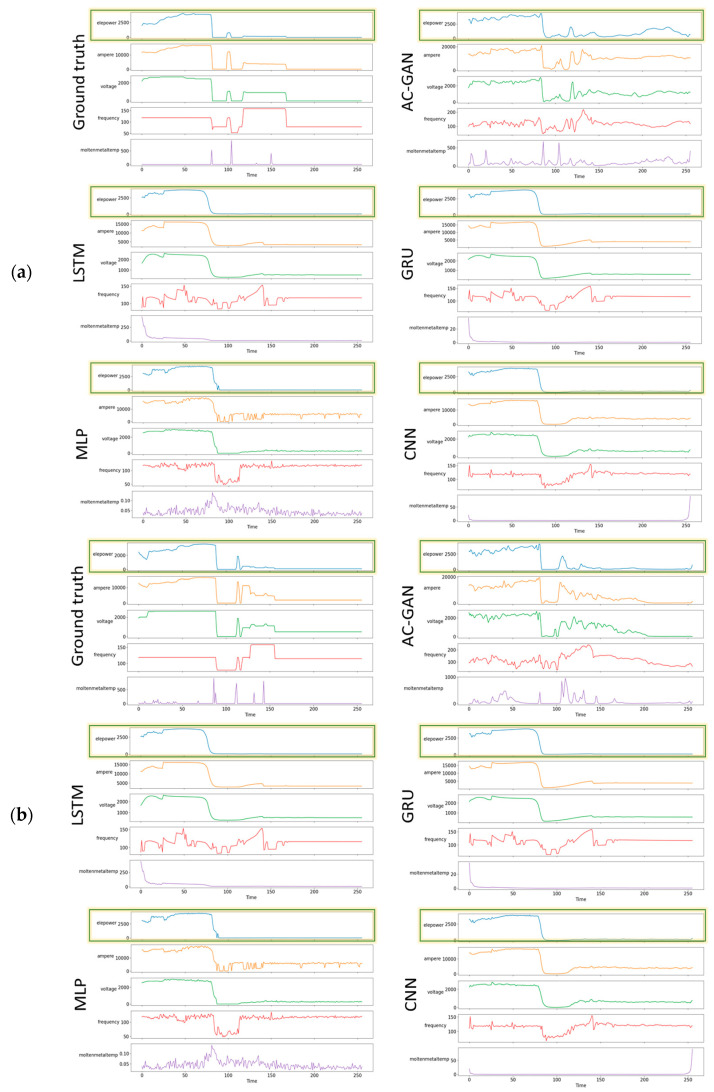

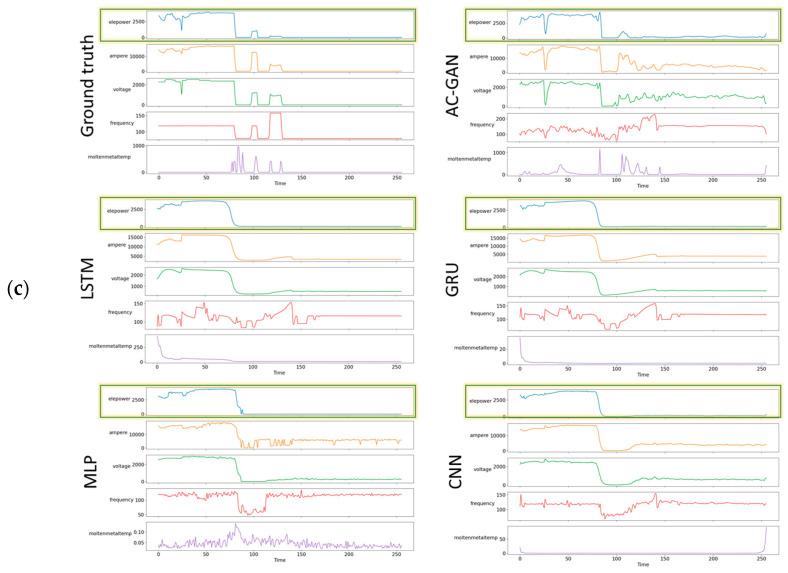
Visual comparison of melting process results: (**a**) input product type p1, (**b**) input product type p3, (**c**) input product type p6.

The main advantage of AC-GAN is that it can generate different data for different inputs. Figure 14 shows a visual comparison of the melting process results. It can be seen that the AC-GAN method generates different TSWP for different inputs, whereas the other models predict the same TSWP for different inputs. The highlighted part of the plots is the electric power variable, which is the control variable of the melting process.

**Figure 15:**
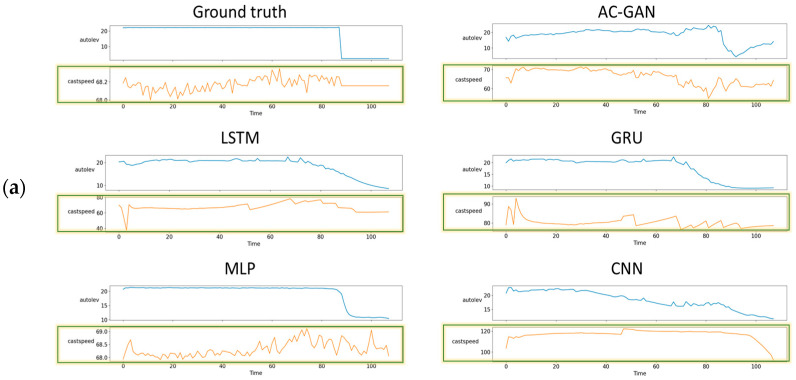

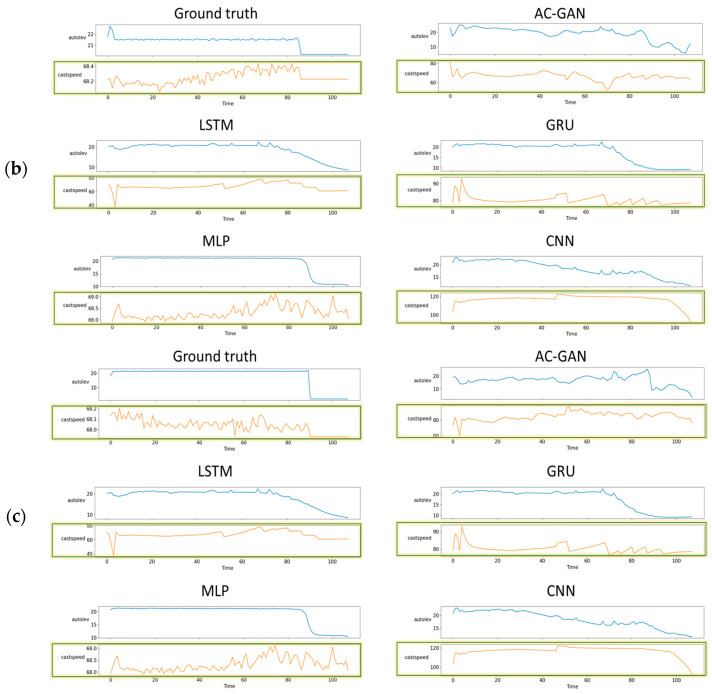
Visual comparison of casting process results: (**a**) input product type p1, (**b**) input product type p3, (**c**) input product type p6.

Figure 15 shows a visual comparison of the casting process results. It can be seen that the AC-GAN also generates different TSWP for different inputs, and the other methods predict the same TSWP for different inputs. The cast speed variable is highlighted as the control variable for the casting process. For both processes, the AC-GAN methods yield better results for different inputs, and the performance metrics indicate that the AC-GAN methods are not overfitted and can generate valuable TSWP even for the new input.

## 5. Conclusions

This study has proposed the generation of TSWP for melting and casting processes using a generative learning approach. The proposed models receive as input categorical data regarding the product type and continuous data regarding material information, depending on the process. The generated data appear noisy, but the key feature of the generated data is different depending on the input data. The other methods based on autoencoder models cannot produce such results. Even though the performance metrics were satisfactory for autoencoder-based models, these models have the same output for different input values. By using the generated data, controllable operation-variable working patterns can be followed so that high-quality products may be manufactured. However, some products have fewer instances than others, and generating such products may be a problem, which is a minor limitation of this research.

In future research, we will apply our method to the system of a manufacturer to aid operators in selecting better working patterns and improving product quality. Consequently, raw material information and ingredient information can be explored further to understand their influence on product quality. In addition, other performance metrics for time-series data can be explored and applied to generative models.

## Figures and Tables

**Table 1:** Real-time sample data.

Time	Electric Power (kW)	Current (A)	Voltage (V)	Frequency (Hz)	Molten Metal Temp. (°C)	Auto Level 1	Auto Level 2	Cast Speed (m/min)
28 April 2019 03:29	1603.631	13,215.75	1728.342	118.5624	0	0	0	0.544504
28 April 2019 03:30	1654.445	13,283.57	1785.511	118.586	0	0	0	0.526617
28 April 2019 03:31	1654.862	13,277.32	1785.814	118.5019	0	0	0	0.515043
28 April 2019 03:32	1653.959	13,269.91	1784.986	118.6013	0	0	0	0.543978
28 April 2019 03:33	1653.589	13,309.26	1785.321	118.5899	1312.96	0	0	0.491895

**Table 2:** Extracted TSWP for melting process.

Time	Electric Power (kW)	Current (A)	Voltage (V)	Frequency (Hz)	Molten Metal Temp. (°C)	Charge#	Lot#
28 April 2019 03:29	1603.631	13,215.75	1728.342	118.5624	0	1	1
28 April 2019 03:30	1654.445	13,283.57	1785.511	118.586	0		
28 April 2019 03:31	1654.862	13,277.32	1785.814	118.5019	0		
28 April 2019 03:32	1653.959	13,269.91	1784.986	118.6013	0		
28 April 2019 03:33	1653.589	13,309.26	1785.321	118.5899	1312.96		

**Table 3:** Extracted TSWP for casting process.

Time	Auto Level	Cast Speed (m/min)	Charge#	Lot#
28 April 2019 03:29	0	0.544504	1	1
28 April 2019 03:30	0	0.526617		
28 April 2019 03:31	0	0.515043		
28 April 2019 03:32	0	0.543978		
28 April 2019 03:33	0	0.491895		

**Table 4:** Statistical summary of data from melting and casting processes.

Stats	Melting Process					Casting Process	
	Electric Power	Current	Voltage	Frequency	Molten Metal Temp.	Auto Level	Cast Speed
Count	802,569	802,569	802,569	802,569	802,569	802,569	802,569
Mean	861.81932	5726.0772	848.32506	91.467603	47.89989	5.055849	18.09078
Std	1413.053	6412.5779	985.21228	45.932171	176.60719	9.030655	31.79986
Min	0	0	0	0.007639	0	0	0.34722
25%	1.388889	13.888889	5.72917	53.022801	0	0	0.526617
50%	97.316385	3082.8703	529.46239	118.48553	0	0	0.543982
75%	988.3102	11,525.926	1321.9415	118.60775	0	1.181452	0.715042
Max	4800	24,000	3300	264	1600	802,569	802,569

**Table 5:** Hyperparameter settings of the methods under comparison.

Parameters/Methods	AC-GAN	MLP	CNN	LSTM	GRU
Optimizer	ADAM	ADAM	ADAM	ADAM	ADAM
Loss	MAE	MAE	MAE	MAE	MAE
Learning_rate	0.0002	0.0002	0.0002	0.0002	0.0002
Beta_1	0.5	0.9	0.99	0.5	0.5

**Table 6:** Comparison of execution times of models.

AC-GAN	MLP	CNN	LSTM	GRU
Execution time for melting models				
1 min 45 s	1 min 33 s	1 min 19 s	1 min 15 s	1 min 17 s
Execution time for casting models				
1 min 24 s	1 min 5 s	58 s	55 s	57 s

**Figure A1:**
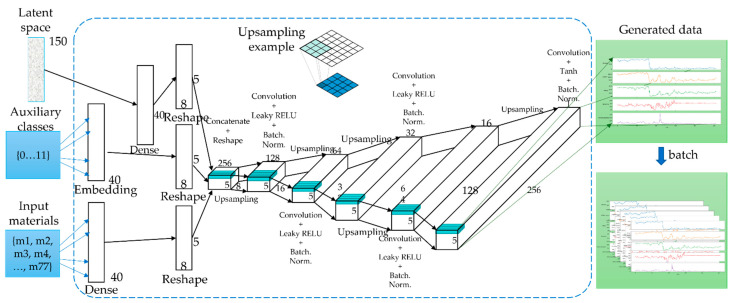
Implementation details of the generator model of AC-GAN for the melting process.

**Figure A2:**
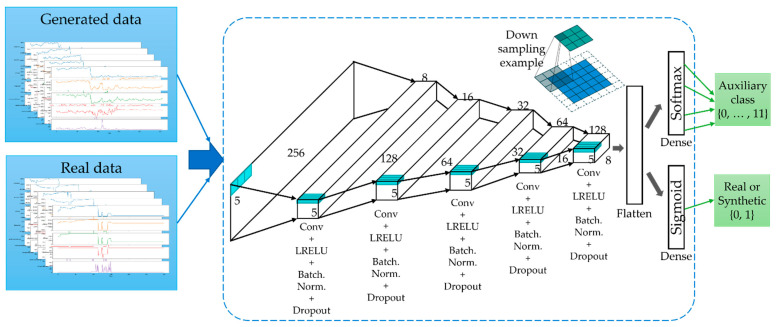
Implementation details of the discriminator model for the melting process.

**Figure A3:**
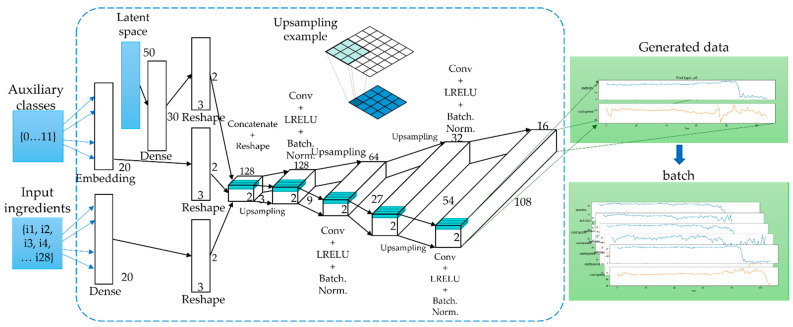
Implementation details of the generator model for the casting process.

**Figure A4:**
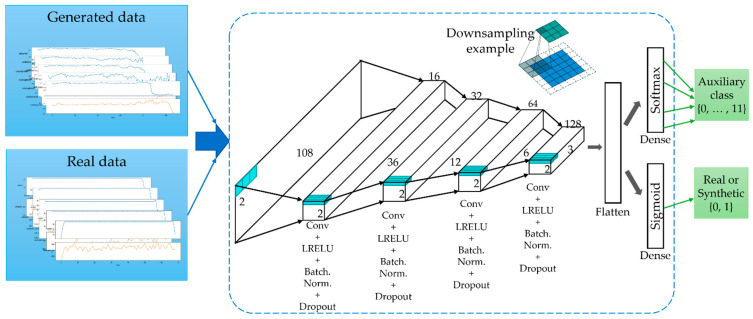
Implementation details of the generator model for the casting process.

## Data Availability

Not applicable.

## Appendix A

In this section, we explain the implementation details of the proposed AC-GAN model. Recall from Section 3.3 that, considering that the input layers have different shapes, we should first pass the data to the dense layers with the same units and reshape them into the same shapes. The reshaped input layers are merged using the concatenate layer of the Keras module. The merged layer is passed to the deconvolutional layer using the Conv2DTranspose layer of Keras, which moves in the opposite direction to a normal convolution. Instead of downsampling the input, it performs upsampling. The output of the deconvolutional layer is passed to the Batch Normalization layer, which passes its output to the Rectified Linear Activation (LeakyReLU) layers. The steps above, starting from the deconvolutional to the LeakyReLU layer, are repeated five times, with different filter sizes for the deconvolutional layers. The last layer is the convolutional layer with filter size 1, activation function tanh, and the same output shape as in the TSWP of the melting process. The details of the implemented input, hidden, and output layers are shown in Figure A1.

The discriminator also consists of an input layer that receives the TSWP of the melting process, which has a shape of 256 × 5. Then, the convolutional layer receives the data from the input and downsamples the input using a stride parameter of 2 × 1. Its output is then passed to the Batch Normalization layer, which passes its output to the LeakyReLU activation layer. Then, the output of the activation layer is passed to the dropout layer at a rate of 0.5. The steps that start from the convolutional layer to the dropout layer are repeated five times by increasing the number of filters in the convolutional layer twice. In the next step, the result of the last layer is passed to the flattened layer of the Keras module. The output of the flattening layer is passed to the first output layer, which is a dense layer with a unit value of 1 and a sigmoid activation function; the second output layer is also a dense layer with a unit value of 11 (distinct number of product types) and a softmax activation function. Figure A2 shows the details of the implemented layers of the discriminator of the melting process. The discriminator model was compiled using the ADAM optimizer with a learning rate of 0.00015 and beta_1 set to 0.5. The loss functions for the outputs are binary_crossentropy for the first output layer and sparse_categorical_crossentropy for the second output layer.

Similar to melting process, we need to reshape the data for casting process. The reshaped layers are joined using the concatenate layer of the Keras module. Then, the result of the concatenated layer is passed to the deconvolutional layer, which passes its output to the Batch Normalization layer. After Batch Normalization, the output of the layer is passed to the LeakyReLU layer. The steps starting from the deconvolutional layer to the LeakyReLU layer are repeated four times because the shape of the TSWP for the casting process is smaller than that for the melting process. The last layer is the same as in the melting process, with the only difference being the output shape, which is the same as in the TSWP for the casting process. Figure A3 shows the details of the generator model that has implemented input, hidden, and output layers.

The discriminator of the casting process consists of an input layer that receives the TSWP of the casting process, which has a shape of 108 × 2. Then, the convolutional layer receives input data and downsamples them using a stride parameter of 2 × 1. Its output is then passed to the Batch Normalization layer, which passes its output to the LeakyReLU activation layer. Then, the output of the activation layer is passed to the dropout layer at a rate of 0.5. The steps that start from the convolutional layer to the dropout layer are repeated four times by increasing the number of filters in the convolutional layer twice. In the next step, the output of the last layer is passed to the flattened layer of the Keras module. The output of the flattening layer is passed to the first output layer, which is a dense layer with a unit value of 1 and a sigmoid activation function; the second output layer is also a dense layer with a unit value of 11 (distinct number of product types) and a Softmax activation function. We note that the discriminator model of the casting process is almost the same as the discriminator of the melting process. Figure A4 shows the details of the implemented layers of the discriminator of the TSWP for the casting process. The discriminator model was compiled using the ADAM optimizer with a learning rate of 0.0001 and beta_1 set to 0.5. The loss functions for the outputs are binary cross-entropy for the first output layer and sparse_categorical_crossentropy for the second output layer.
